# The Synthetic Dipeptide Pidotimod Shows a Chemokine-Like Activity through CXC Chemokine Receptor 3 (CXCR3)

**DOI:** 10.3390/ijms20215287

**Published:** 2019-10-24

**Authors:** Francesca Caccuri, Antonella Bugatti, Silvia Corbellini, Sara Roversi, Alberto Zani, Pietro Mazzuca, Stefania Marsico, Arnaldo Caruso, Cinzia Giagulli

**Affiliations:** 1 Section of Microbiology, Department of Molecular and Translational Medicine, University of Brescia, 25123 Brescia, Italy; francesca.caccuri@unibs.it (F.C.); antonella.bugatti@unibs.it (A.B.); s.roversi003@unibs.it (S.R.); a.zani033@unibs.it (A.Z.); p.mazzuca@unibs.it (P.M.); arnaldo.caruso@unibs.it (A.C.); 2 Laboratory of Microbiology and Virology, Azienda Socio Sanitaria Territoriale Spedali Civili, 25123 Brescia, Italy; silvia.corbellini@gmail.com; 3 Department of Pharmacy, Health and Nutritional Sciences, University of Calabria, Arcavacata di Rende, 87036 Cosenza, Italy; stefania.marsico@unical.it

**Keywords:** pidotimod, CXCR3, monocyte, migration, PI3K/Akt pathway, T cell, immunomodulant

## Abstract

In recent years immunomodulators have gained a strong interest and represent nowadays an active expanding area of research for the control of microbial diseases and for their therapeutic potential in preventing, treating and reducing the morbidity and mortality of different diseases. Pidotimod (3-L-pyroglutamyl-L-thiaziolidine-4carboxylic acid, PDT) is a synthetic dipeptide, which possesses immunomodulatory properties and exerts a well-defined pharmacological activity against infections, but its real mechanism of action is still undefined. Here, we show that PDT is capable of activating tyrosine phosphorylation-based cell signaling in human primary monocytes and triggering rapid adhesion and chemotaxis. PDT-induced monocyte migration requires the activation of the PI3K/Akt signaling pathway and chemokine receptor CXCR3. Indeed, a mAb to CXCR3 and a specific receptor inhibitor suppressed significantly PDT-dependent chemotaxis, and CXCR3-silenced primary monocytes lost responsiveness to PDT chemoattraction. Moreover, our results highlighted that the PDT-induced migratory activity is sustained by the CXCR3A isoform, since CXCR3-transfected L1.2 cells acquired responsiveness to PDT stimulation. Finally, we show that PDT, as CXCR3 ligands, is also able to direct the migration of IL-2 activated T cells, which express the highest levels of CXCR3 among CXCR3-expressing cells. In conclusion, our study defines a chemokine-like activity for PDT through CXCR3A and points on the possible role that this synthetic dipeptide may play in leukocyte trafficking and function. Since recent studies have highlighted diverse therapeutic roles for molecules which activates CXCR3, our findings call for an exploration of using this dipeptide in different pathological processes.

## 1. Introduction

Viruses are the main agents responsible for Acute Respiratory Tract Infections (ARTIs) during the pediatric age [1]. The introduction of new antibiotics and vaccines has surely contributed to control the most life-threatening ARTIs but has not had a strong impact on viral ARTIs. Therefore, one efficient approach in preventing and treating ARTIs is to increase the immune response by enhancing the child’s innate defense mechanisms. The last decade has seen the emergence of different kinds of natural and synthetic molecules with different mechanisms of action, called immunomodulators, which have been introduced for prophylaxis and treatment of various infectious diseases and inflammation [2,3,4,5,6]. Some of these substances are granulocyte colony-stimulating factor (G-CSF), interferons, imiquimod and bacterial-derived preparations, which are already licensed for use in patients. Others including IL-12, various chemokines, synthetic cytosine phosphate-guanosine (CpG) oligodeoxynucleotides and glucans, have been investigated extensively in clinical and preclinical studies [7]. Another compound, which exerts a well-defined immunomodulatory and antimicrobial activity against infections is Pidotimod (3-L-pyroglutamyl-L-thiaziolidine-4carboxylic acid, PDT) [8,9], a synthetic dipeptide on which research focused particular attention for its properties in prevention and treatment of ARTIs in childhood. Indeed, different studies have shown that PDT exerts a beneficial effect in children reducing the number of ARTIs, the severity of signs and symptoms of acute episodes [8,10,11,12,13,14].

In vitro studies, performed using either murine or human cells, have shown that PDT is able to modulate both innate and adaptive immune responses [15]. In particular, this dipeptide upregulates the expression of HLA-DR and of the co-stimulatory molecules CD83 and CD86 on dendritic cells (DCs) inducing their maturation. It also stimulates DCs to release pro-inflammatory molecules, stimulates natural killer cell activity, inhibits thymocyte apoptosis and potentiates phagocytosis [16,17,18,19]. This dipeptide has also been described to enhance the proliferation of mitogen-activated peripheral blood mononuclear cells (PBMCs) and increase the production of cytokines, as IFN-α, IFN-γ and IL-12, crucial to drive T cell proliferation and differentiation towards a Th1 phenotype [16,19,20]. More recently, Fogli et al. (2014) showed that the treatment of PBMCs and monocytes with PDT led to a significant attenuation of the inflammatory response to TLR agonists. Since TLRs are key components in pathogen recognition and critical mediators in the early response to foreign microorganisms, attenuation of the inflammatory response to TLR agonists by PDT represents an important immunomodulating effect [21].

Currently, the research focused on this molecule has attempted to better elucidate its mechanism of action. The ability of PDT to modulate different aspects of both innate and adaptive immune response leaves to speculate that this dipeptide might exert its function through the involvement of one or more cytokine/chemokine receptors. Indeed, these receptors play an essential role in the immune response and are responsible for activation and recruitment of immune cells at infection and inflammation sites, contributing to the induction and exacerbation of chronic inflammatory reactions. However, despite the numerous efforts, there are limited information about the cellular receptor(s) engaged on immune cells and intracellular signaling pathways triggered by this molecule. Currently, a new research input could be now essential on the role of PDT as immunomodulant, since immunomodulation represents an adjunct modality which looks promising for control of microbial diseases and in the future could play a key role in treating and reducing the morbidity and mortality of different diseases.

In this study we investigated the PDT mechanism of action, thus, to better understand how this dipeptide exerts its biological activities and the why of its efficacy and safety.

Here, we demonstrate that PDT shows a chemokine-like activity through the activation of CXCR3 receptor, in particular CXCRA isoform, and of the PI3K/Akt signaling pathway.

## 2. Results

PDT activates rapid intracellular tyrosine phosphorylation-based protein signaling on human primary monocytes.

Rapid phosphorylation of signaling proteins is triggered by immune cell surface receptors and plays a pivotal role in the regulation of innate and adaptive immune functions. In particular, tyrosine phosphorylation (pTyr) of signaling proteins is indispensable in the regulatory pathways and represents a key activation signal, promoted by cytokine/chemokine receptors [22,23,24]. Therefore, in order to understand if PDT is able to trigger a cytokine/chemokine receptor, we investigated its capability to activate in a few min tyrosine phosphorylation-based cell signaling on human primary monocytes.

**Figure 1:**
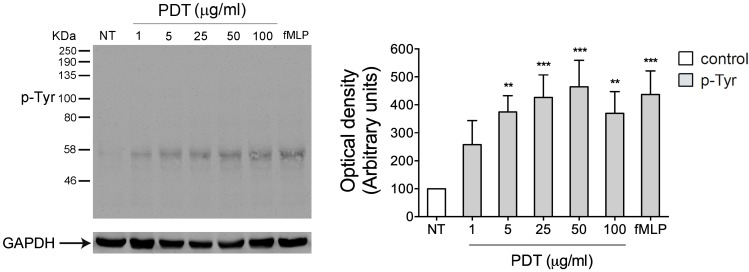
Effect of PDT stimulation on protein tyrosine phosphorylation in monocytes. Monocytes were treated for 5 min with 1, 5, 25, 50, 100 μg/mL of PDT and 10 nM of fMLP. Untreated cells were used as control (NT). Western blot analysis of cells lysates shows that PDT is able to induce an increase of Tyr phosphorylated proteins at different concentrations tested, as shown by densitometry analysis and plotting of the pTyr/GAPDH. In the left panel blots from one representative experiment of three with similar results are shown. In the right panels, values reported for protein Tyr phosphorylation are the mean ± SD of three independent experiments. Statistical analysis was performed by one-way ANOVA and the Bonferroni’s post-test was used to compare data, ** *p <* 0.01, *** *p* < 0.001.

To this aim monocytes were stimulated at 37 °C for 5 min with the dipeptide PDT at different concentrations (1, 5, 25, 50, 100 μg/mL) and the tripeptide fMLP (10 nM), used as positive control. Data obtained from western blot analysis, using a protein specific anti-phosphotyrosine antibody, show that extracts from untreated cells have a low basal level of Tyr phosphorylated proteins. At the same time, PDT dose-response analysis shows that the immunomodulant is able to trigger a rapid increase of Tyr phosphorylated proteins at concentrations ranging from 5 to 100 μg/mL (Figure 1). As expected, fMLP induced a significant level of Tyr phosphorylated proteins.

These data show the PDT ability to induce protein tyrosine phosphorylation in monocytes and suggest the capability of the dipeptide to act probably through a cytokine/chemokine receptor activation.

### 2.1. PDT Induces Monocyte Adhesion and Migration

**Figure 2:**
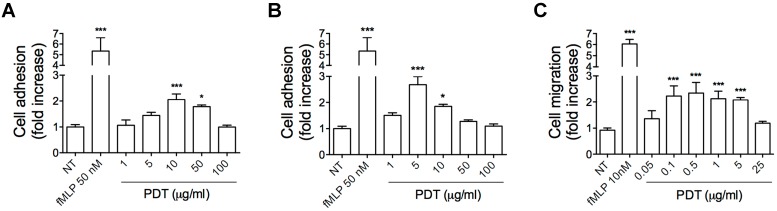
Effect of PDT on monocytes adhesion and migration. (**A**,**B**) Static adhesion assay on ICAM-1 (**A**) and VCAM-1 (**B**). Monocytes were stimulated or not (NT) for 2 min at 37 °C with PDT at the indicated concentrations. Bars represent the means ± SD of 3 independent experiments performed in triplicate. Statistical analysis was performed by one-way ANOVA and the Bonferroni’s post-test was used to compare data, *** *p* < 0.001, * *p* < 0.05. (**C**) Transwell migration assays of monocytes in response to the indicated treatments. Bars represent the means ± SD of 3 independent experiments performed in triplicate. Statistical analysis was performed by one-way ANOVA and the Bonferroni’s post-test was used to compare data, *** *p* < 0.001. NT = not treated.

Chemokines, through chemokine receptor activation, trigger intracellular signaling events, which control leukocyte recruitment, a key multi-step process in regulation of immune responses involving rapid integrin-dependent adhesion and migration of leukocytes [25]. In order to assess the ability of PDT to functionally activate a chemokine receptor on monocytes, we performed static adhesion and migration assays. Static adhesion assays were performed on immobilized ligands, as ICAM-1 and VCAM-1, in response to different concentration of the synthetic dipeptide (1, 5, 10, 50, 100 μg/mL). Figure 2 shows that PDT triggered a rapid (2 min) concentration-dependent adhesion of primary human monocytes to ICAM-1 (Figure 2A) and V-CAM (Figure 2B). In particular, PDT significantly stimulated monocyte adhesion on ICAM-1 at a concentration ranging from 10–50 μg/mL with a peak at 10 μg/mL (Figure 2A). On the other hand, PDT-induced adhesion on VCAM-1 occurred at a lower concentration, ranging from 5 to 10 μg/mL and reaching a peak at 5 μg/mL (Figure 2B).

Then, we performed monocyte migration in Transwell chemotaxis assays in response to different concentrations of the dipeptide (0.05, 0.1, 0.5, 1, 5, 25 μg/mL). In Figure 2C we show that PDT stimulates chemoattraction of monocytes at a concentration ranging between 0.1 and 5 μg/mL.

These data show that monocyte adhesion requires a higher PDT concentration than that required for chemotaxis. This phenomenon is common to chemokines and can be elucidated by the findings of Campbell et al. (1996), who demonstrated that adhesion requires a high agonist concentration with the simultaneous occupancy of many receptors, whereas chemotaxis occurs at low agonist concentration. These different requirements for triggering adhesion and chemotaxis are necessary for their independent regulation [26].

Overall, these results show the capability of PDT to stimulate rapid adhesion and migration of human primary monocytes, suggesting a chemokine-like role for the dipeptide and its ability to transduce, through a chemokine receptor, intracellular signals involved in regulation of cell motility.

### 2.2. PTx Treatment Inhibits PDT-Induced Chemokine Activity and Tyrosine Phosphorylation-based Protein Signaling in Monocytes

Chemokines bind and signal through seven-transmembrane receptors coupled with the Gi class of heterotrimeric G proteins. Pertussis toxin (PTx) is known to prevent the Gi proteins interaction with G protein–coupled receptors, thus blocking intracellular signaling cascade. In order to determine if PDT receptor is coupled to Gi proteins, monocytes were pretreated with 500 ng/mL of PTx for 2 h at 37 °C, then stimulated with the dipeptide and tested for their capability to adhere, migrate and trigger phosphorylation-based cell signaling.

**Figure 3:**
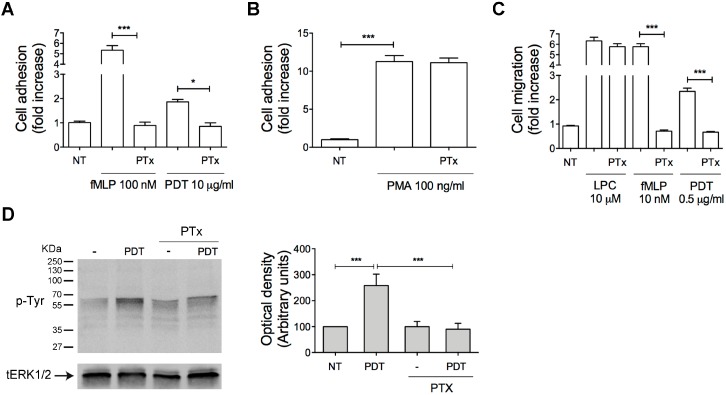
Effect of PTx on PDT-induced monocytes adhesion, migration and protein tyrosine phosphorylation. (A, B) Static adhesion assay of monocytes on ICAM-1. Cells pretreated with 500 ng/mL of PTx for 2 h at 37 °C were stimulated for 2 min at 37 °C with PBS (NT), fMLP (100 nM), PDT (10 µg/mL) (**A**) or for 10 min with PMA (100 ng/mL) (**B**). Bars represent the means ± SD of 3 independent experiments performed in triplicate. Statistical analysis was performed by paired 2-tail Student *t* test, *** *p* < 0.001, * *p* < 0.05. (**C**) Transwell migration assay of monocytes in response to the indicated treatments. Monocytes pretreated with PTx were stimulated for 90 min at 37 °C with PBS (NT), LPC (10 µM), fMLP (10 nM) or PDT (0.5 µg/mL). Bars represent the means ± SD of 3 independent experiments performed in triplicate. Statistical analysis was performed by paired 2-tail Student *t* test, *** *p* < 0.001. (**D**) Monocytes pretreated or not with PTx were stimulated or not for 5 min at 37 °C with PDT (5 µg/mL). Western blot analysis of cell lysates shows that PDT-triggered protein tyrosine phosphorylation was inhibited by PTx, as shown by densitometry analysis and plotting of the pTyr/tERK1/2. In the left panel blots from one representative experiment of three with similar results are shown. In the right panels, values reported for protein Tyr phosphorylation are the mean ± SD of three independent experiments. Statistical analysis was performed by paired 2-tail Student *t* test and the Bonferroni’s post-test was used to compare data, *** *p* < 0.001. NT = not treated.

PDT-triggered monocyte adhesion on ICAM-1 and migration were completely inhibited by PTx pretreatment (Figure 3A and 3C, respectively). In the same assays, as expected, the strong adhesion and chemotaxis induced by fMLP (100 nM and 10 nM, respectively), a reference chemoattractant able to transduce intracellular signals through Gi proteins, was inhibited by PTx (Figure 3A,C) [26,27,28]. The inhibitory effect of PTx was not attributable to a generic toxic effect of PTx pretreatment, because adhesion of monocytes was retained after 10 min of PMA (phorbol 12-myristate 13-acetate) stimulation (100 ng/mL) (Figure 3B), and cell migration occurred in response to LPC (L-α-lysophosphatidylcholine, palmitoyl C16:0, Sigma-Aldrich) (10 µM) (Figure 3C), in line with previous observations [27,29,30].

In addition, to determine if rapid tyrosine phosphorylation-based protein signaling triggered by the dipeptide was dependent from a receptor coupled with Gi proteins, monocytes were pretreated with PTx, then stimulated with PDT (5 μg/mL) and tested for their capability to induce protein tyrosine phosphorylation. Data obtained from western blot analysis, using a protein specific anti-phosphotyrosine antibody, showed that PDT-triggered protein tyrosine phosphorylation was completely inhibited by PTx pretreatment (Figure 3D).

Overall, these data showed that PDT induces monocyte adhesion, migration and intracellular protein tyrosine phosphorylation through a cell surface receptor coupled with the Gi class of heterodimeric G proteins.

### 2.3. Monocyte Migration Triggered by PDT Requires the Activation of PI3K/Akt Signaling Pathway

**Figure 4:**
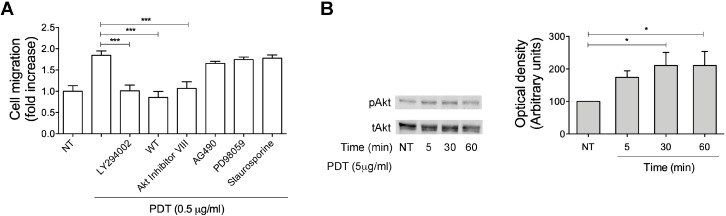
Signaling molecules involved in PDT-triggered monocyte migration. (**A**) Transwell migration assay of monocytes in response to the indicated treatments. Monocytes pretreated for 1 h at 37 °C with the inhibitors LY294002 (25 μM), wortmannin (100 nM), Akt inhibitor VIII (1 μM), AG490 (100 μM), PD98059 (10 μM) and staurosporine (1 nM) were stimulated for 90 min at 37 °C with PBS (NT) or PDT (0.5 µg/mL). Bars represent the means ± SD of 3 independent experiments performed in triplicate. Statistical analysis was performed by one-way ANOVA and the Bonferroni’s post-test was used to compare data, *** *p* < 0.001. (**B**) Monocytes were stimulated with 5 µg/mL of PDT at 37 °C for the indicated times. Not treated cells (NT) were used as control (lane 1). Western blot analysis of monocyte lysates shows that PDT activates Akt, as shown by the respective phosphorylation state, verified by densitometric analysis and plotting of the phospho-Akt/total Akt (pAkt/tAkt). In the left panel blots from one representative experiment of three with similar results are shown. In the right panel, values reported for Akt phosphorylation are the mean ± SD of three independent experiments. Statistical analysis was performed by one-way ANOVA and the Bonferroni’s post-test was used to compare data, * *p* < 0.05.

Cell migration is governed by a complex network of signal transduction pathways, which involve lipid second messengers, small GTPases, kinases, cytoskeleton-modifying proteins, and culminates in the cytoskeletal remodeling and chemoattraction response to ligand-induced receptor activation [31]. In order to investigate the possible signaling pathways responsible for monocyte recruitment triggered by PDT stimulation, primary human monocytes were pretreated for 60 min at 37 °C with inhibitors of different key signaling molecules and then stimulated with the dipeptide (0.5 μg/mL) to migrate in a Transwell chemotaxis system. As shown in the Figure 4A, AG490 (10 μM), a specific and potent inhibitor of the Janus kinase 2 protein (JAK2), as well as PD98059 (10 μM), a MAP kinase/extracellular signal-regulated kinase (ERK) inhibitor, and staurosporine, a strong inhibitor of Protein Kinase C (PKC) and other protein kinases, did not impact on monocyte migration induced by PDT (Figure 4A). On the other hand, PDT-triggered monocyte migration was completely inhibited by two specific inhibitors of PI3K (wortmannin 100 nM and LY294002 10 μM) and by the specific Akt inhibitor VIII (1 μM) (Figure 4A). Therefore, in order to confirm the capability of PDT to activate PI3K/Akt signaling pathways, we assessed by western blot analysis the Akt phosphorylation status at Ser473 in cytosolic extracts of cells, treated for 5, 30 and 60 min with the dipeptide (5 μg/mL). As shown in Figure 4B, monocytes treated with PDT (0.5 g/mL) for 30 min showed a significant Akt phosphorylation, which remained sustained up to 60 min.

Overall, these data show that PDT-induced monocyte chemotaxis is due to the activation of the PI3K/Akt signaling pathway, which is known to be implicated in cell migration and in downstream signaling of cytokine/chemokine receptors [32,33].

### 2.4. PDT-Triggered Monocyte Migration Is Mediated by CXCR3

**Figure 5:**
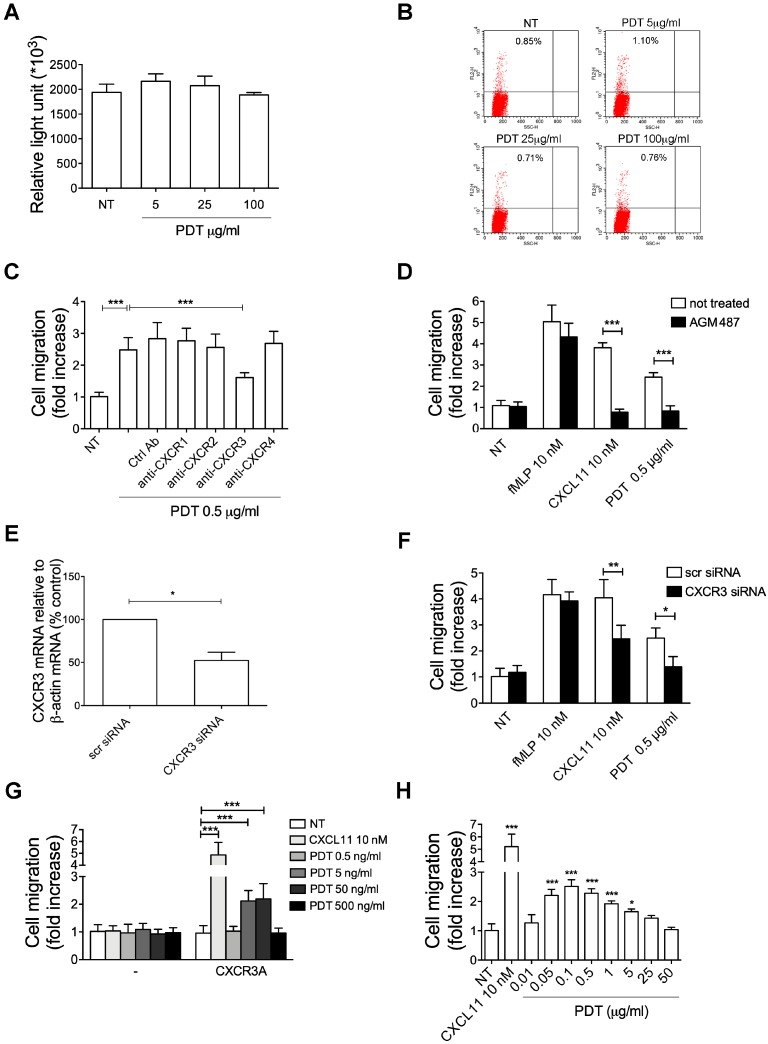
PDT–induced chemotactic activity is mediated by CXCR3. (**A**) Cells were treated or not (NT) with PDT (5, 25, 100 μg/mL) for 2 h. Cell viability was assessed on 10^3^ cells by CellTiter-Glo^®^ Luminescent Cell Viability Assay, based on determination of intracellular ATP concentration, according to manufacturer’s instructions. Bars represent the means ± SD of 3 independent experiments. (**B**) Cells were treated or not (NT) with PDT (5, 25, 100 μg/mL) for 2 h, stained with propidium iodide and analyzed by flow cytometer. Data were analyzed by CellQuest software. Figures are representative of one experiment of three with similar results. (**C**) Transwell migration assay of monocytes in response to the indicated treatments. Monocytes pretreated for 1 h at 37 °C with 50 µg/mL of Ctrl mAb or mAb to anti-CXCR1 or anti-CXCR2 or anti-CXCR3 or anti-CXCR4 were stimulated for 90 min at 37 °C with PBS (NT) or PDT 0.5 µg/mL. Bars represent the mean ± SD of three independent experiments performed in triplicate. Statistical analysis was performed by one-way ANOVA and the Bonferroni’s post-test was used to compare data, *** *p* < 0.001. (**D**) Transwell migration assay of monocytes pretreated for 1 h at 37°C with the inhibitor AGM487 (0.5 μM) and stimulated for 90 min at 37 °C with PBS (NT), fMLP (10 nM) or CXCL11 (10 nM) or PDT (0.5 µg/mL). Bars represent the means ± SD of 3 independent experiments performed in triplicate. Statistical analysis was performed by paired 2-tail Student *t* test, *** *p* < 0.001. (**E**) Analysis of CXCR3 gene expression performed using quantitative real time PCR. Monocytes were nucleoporated with scrambled siRNAs, used as negative control or with a pool of four distinct siRNAs specific for four distinct regions of CXCR3. Analysis of real time PCR data was performed with the 2^−ΔΔ^*^C^*^t^ method using Relative Quantitation Study software. Quantification of CXCR3 mRNA was normalized in each reaction according to the internal β-actin control. Bars represent the mean ± SD of three independent experiments performed in triplicate. (**F**) Transwell migration assay of monocytes nucleoporated with specific siRNAs for CXCR3 or with scrambled fluorescein-labeled siRNAs (scr siRNA), used as negative control, in response to the indicated treatments. Bars represent the mean ± SD of three independent experiments performed in triplicate. Statistical analysis was performed by paired two-tail Student’s *t*-test, ** *p* < 0.01, * *p* < 0.05. NT = not treated (**G**) Transwell migration assay of L1.2 cells transfected with pTarget empty vector (-) or expressing CXCR3A and stimulated for 90 min at 37°C with PBS (NT), CXCL11 (10 nM) and PDT (0.5, 5, 50, 500 ng/mL). Bars represent the mean ± SD of three independent experiments performed in triplicate. Statistical analysis was performed by two-way ANOVA. Bonferroni’s post-test was used to compare data, *** *p* < 0.001. (**H**) Transwell migration assay of IL-2-activated T lymphocytes in response to the indicated treatments. Bars represent the means ± SD of 3 independent experiments performed in triplicate. Statistical analysis was performed by one-way ANOVA and the Bonferroni’s post-test was used to compare data, * *p* < 0.05, *** *p* < 0.001.

Purinergic receptors are G protein-coupled receptors (GPCR) involved in several cellular functions, including cell migration and its function is mediated by Gi proteins [34]. So, in order to exclude the activation of purinergic receptors from the nucleotide release due to a PDT cytotoxic effect, we evaluate the viability of monocytes treated for 2h with different concentrations of PDT (5, 25, 100 μg/mL) by CellTiter-Glo® Luminescent Cell Viability Assay, which is based on determination of intracellular ATP concentration, as indicator of metabolically active cells, and propidium iodide, which can stain nucleic acids inside of dead or damaged cells. As shown in Figure 5A,B, the cells were metabolically active at any tested concentration. Therefore, we can exclude a purinergic receptor activation by the dipeptide.

Human monocytes express numerous chemokine receptors, among which CXCR1, CXCR2, CXCR3, CXCR4 might play a role in PDT chemokine-like activity. Indeed, these receptors are coupled with the Gi class of G proteins and involved in promoting monocyte migration [35,36,37]. Therefore, first we tested whether PDT-triggered monocyte migration could be ascribed to the activation by the dipeptide of one of these receptors.

To this aim we performed Transwell chemotaxis assays with monocytes pre-treated for 1 h at 37 °C with neutralizing mAbs to CXCR1, CXCR2, CXCR3, CXCR4 or with a control mAb. The specificity of the antibodies is reported in Supplementary Material (Figure S1). As shown in Figure 5C, mAb to CXCR3 significantly inhibited PDT-dependent chemotaxis. Moreover, no inhibition of PDT activity was observed in monocytes pre-treated with the control mAb or with mAbs to CXCR1, CXCR2 and CXCR4.

In order to confirm the involvement of CXCR3 in monocyte migration induced by PDT, we also performed chemotaxis assays with cells pre-treated 36 h at 37 °C with the CXCR3 antagonist AMG487 (0.5 μM). As expected, the antagonist was able to significantly inhibit monocyte migration triggered by the dipeptide (Figure 5D). The specific effect of AMG487 was confirmed by the fact that fMLP-induced monocyte migration (10 nM) was not influenced by the presence of this antagonist, and on the other side, CXCL11-stimulated chemotaxis (10 nM), known to be mediated by CXCR3, was completely inhibited (Figure 5D).

To further assess the involvement of CXCR3 in PDT chemokine activity, the expression of this receptor on monocytes was suppressed by silencing, using the AMAXA nucleofection technology to deliver specific siRNAs. Efficiency of nucleofection was evaluated after 16 h post-nucleofection by flow cytometric analysis and showed an average of 88% ± 8% nucleoporated monocytes. The effects of siRNA on CXCR3 expression were evaluated by real-time PCR analysis. As shown in Figure 5E, approximately 52% inhibition of CXCR3 transcripts was observed in monocytes at 16 h after nucleofection with CXCR3 siRNAs as compared to monocytes nucleofected with scr siRNA. The CXCR3 silencing induced a significant inhibition of PDT-triggered migration of monocytes as compared to cells nucleofected with an unrelated scrambled (scr) siRNAs (Figure 5F). Moreover, as expected, a significant reduction of monocyte migratory activity to CXCL11 (10 nM) (Figure 5F), one of the physiological ligands of CXCR3, confirmed the silencing of the receptor. Finally, the specificity of CXCR3 silencing was confirmed by the finding that fMPL-induced monocyte migration (10 nM) was not influenced by inhibition of transcripts (Figure 5F).

In conclusion, all these data suggest that PDT-triggered migratory activity of PDT is mediated by CXCR3.

### 2.5. The PDT-Induced Migratory Activity Is Sustained by the CXCR3A Isoform

CXCR3 gene is alternatively spliced, generating at least three functional isoforms differing in either their N or C-terminus. The more studied CXCR3 isoforms are CXCR3A and CXCR3B. It’s known that G protein recruitment by CXCR3 isoforms is different and leads to different signaling cascades. Indeed, CXCR3A isoform is linked to Gαi or Gαq proteins, which promote cell migration by activating the PI3K/Akt pathway [38], while CXCR3B variant has been reported to be pertussis toxin-insensitive and possibly Gs coupled [39]. Since we have established that PDT acts as a chemokine in a pertussis toxin-sensitive manner, through activation of PI3K/Akt signaling pathway, we explored if a de novo expression of CXCR3A was responsible for the dipeptide activity. To this aim, L1.2 cells were used to express CXCR3A. Cells were nucleofected with fluorescent positive control vector (pmaxGFPTM Control vector) or pTarget empty vector or expressing human CXCR3A. The efficiency of nucleofection, evaluated by flow cytometry, was found to be of 86% ± 6% in 3 different experiments. Flow cytometric analysis showed that a high percentage of cells nucleofected with pTarget-CXCR3A expressed the receptor on their surface (88% ± 8%), when compared to cells nucleofected with the control empty vector. The migratory behavior of L1.2 cells expressing the empty vector and CXCR3A receptor following CXCL11 (10 nM) or PDT stimulation (0.5, 5, 50, 500 ng/mL) was assessed by Transwell chemotaxis assays. Figure 5G shows that empty vector expression did not induce cells to migrate in response to PDT or CXCL11. On the other hand, expression of CXCR3A was found to significantly promote migration in response to CXCL11, the physiological ligand of this receptor, and PDT. The migratory activity triggered by PDT in CXCR3A expressing L1.2 cells was dose dependent with a peak of activity at the concentrations of 5 and 50 ng/mL.

Therefore, our results show that a de novo expression of CXCR3A in L1.2 cells is sufficient to mediate cell migration in response to PDT stimulation.

### 2.6. Pidotimod Induces Migration of IL-2-Activated T Lymphocytes

Chemokine receptors are specifically and differently expressed on different subsets of leukocytes to control a selective tissue recruitment of functional immune cells [40]. CXCR3 is rapidly induced on resting T cells following activation and plays an important role in T cell trafficking and function. Therefore, in order to confirm a chemokine-like activity for PDT through the CXCR3 receptor, we performed migration assays with a 5-μm Transwell chemotaxis system on IL-2-activated T cells, which express high levels of this receptor on their surface [40], in response to different PDT concentrations (0.01, 0.05, 0.1, 0.5, 1, 5, 25, 50 μg/mL). As expected, PDT stimulated a statistically significant chemoattraction of IL-2-activated T lymphocytes and its activity occurred at a concentration ranging between 0.05 and 5 μg/mL with a bell-shaped chemotactic dose-response curve and a peak activity at 0.1 μg/mL (2.5-fold increase) (Figure 5H). Moreover, as expected, a strong induction of migration was obtained with IL-2-activated T lymphocytes using CXCL11 (10 nM), a reference chemoattractant for CXCR3 receptor (Figure 5H).

Overall, these data show that PDT is able also to stimulate migration of IL-2-activated T lymphocytes.

### 2.7. CXCL11, as PDT, Triggers Monocyte Migration through Akt Kinase Activation

**Figure 6:**
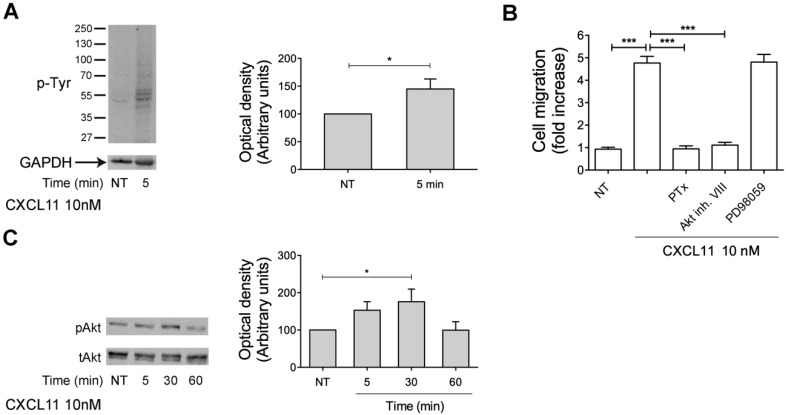
CXCL11–induced chemotactic activity, as PDT one, is mediated by Akt kinase. (**A**) Monocytes were treated or not for 5 min with 10nM of CXCL11. Western blot analysis of cells lysates shows that CXCL11 is able to induce an increase of Tyr phosphorylated proteins, as shown by densitometry analysis and plotting of the pTyr/GAPDH. In the left panel blots from one representative experiment of three with similar results are shown. In the right panels, values reported for protein Tyr phosphorylation are the mean ± SD of three independent experiments. Statistical analysis was performed by Student’s *t*-test, * *p <* 0.05. NT = not treated. (**B**) Transwell migration assay of monocytes in response to the indicated treatments. Monocytes pretreated with PTx (500 ng/mL) or Akt inhibitor VIII (1 μM) or PD98059 (10 μM) were stimulated for 90 min at 37 °C with PBS (NT) or CXCL11 (10 nM). Bars represent the means ± SD of 3 independent experiments performed in triplicate. Statistical analysis was performed by one-way ANOVA and the Bonferroni’s post-test was used to compare data. *** *p* < 0.001. (**C**) Monocytes were stimulated with 10 nM of CXCL11 at 37 °C for the indicated times. Not treated cells (NT) were used as control (lane 1). Western blot analysis of monocyte lysates shows that CXCL11 activates Akt, as shown by the respective phosphorylation state, verified by densitometric analysis and plotting of the phospho-Akt/total Akt (pAkt/tAkt). In the left panel blots from one representative experiment of three with similar results are shown. In the right panel, values reported for Akt phosphorylation are the mean ± SD of three independent experiments. Statistical analysis was performed by one-way ANOVA and the Bonferroni’s post-test was used to compare data, * *p <* 0.05.

Here, we have shown that PDT exerts its chemotactic activity through CXCR3 activation triggering PI3K/Akt signaling pathway. In order to confirm that PDT induces monocyte migration with the same mechanism used by the physiological ligand of CXCR3, CXCL11, we performed monocyte migration assays and western blot analysis. First, monocytes were stimulated or not at 37 °C for 5 min with CXCL11 (10 nM) and data obtained from western blot analysis show that, as expected, CXCL11 is able to trigger a rapid increase of Tyr phosphorylated proteins (Figure 6A). Then, in order to investigate if the PI3K/Akt signaling pathway, responsible for PDT-induced monocyte recruitment, is also triggered by CXCL11, monocytes were pretreated at 37 °C with PTx, Akt inhibitor VIII (1 μM) and MAPK/ERK inhibitor PD98059 (10 μM) and then stimulated with CXCL11 (10 nM) to migrate in a Transwell chemotaxis system. As shown in the Figure 6B, PD98059 did not influence monocyte migration induced by CXCL11, while PTx and the specific Akt inhibitor VIII (1 μM) completely inhibited CXCL11-triggered monocyte migration (Figure 4A). Finally, in order to confirm the capability of CXCL11 to activate Akt kinase, we assessed by western blot analysis the Akt phosphorylation status at Ser473 in cytosolic extracts of monocytes, treated for 5, 30 and 60 min with CXCL11 (10 nM). As shown in Figure 6C, monocytes stimulated with CXCL11 for 30 min showed a significant Akt phosphorylation.

Overall, these data show that CXCL11-induced monocyte chemotaxis, like PDT one, is due to the activation of Akt kinase.

## 3. Discussion

In the present study for the first time we demonstrate that PDT exerts a chemokine-like activity triggering monocyte adhesion and migration. In this regard, PDT can be considered the shortest synthetic peptide known up to date with a chemokine activity.

Our experimental evidences highlight the ability of the dipeptide to functionally activate the G protein-coupled receptor CXCR3, in particular the CXCR3A isoform, which is linked to heterotrimeric GTP-binding proteins of the Gi family. Indeed, PDT-induced chemokine activity was PTx-sensitive and neutralized by an anti-human CXCR3 mAb, a specific CXCR3 inhibitor and CXCR3 siRNA. Moreover, the specificity of CXCR3-mediated effects was confirmed by the capability of dipeptide to chemoattract L1.2 cells stably expressing the human CXCR3A isoform. The study of intracellular signaling downstream of CXCR3 showed that PDT triggers the PI3K/Akt pathway. The activation of such signaling cascade has also been previously observed in response to a wide number of CXCR3 agonists in different cell types and it is in line with the activity previously reported for this canonical receptor [41,42,43].

GPCRs represent one of the therapeutically most relevant protein families and around one-third of the available small-molecule drugs target GPCRs [44]. Surprisingly, PDT, which is simply a dipeptide and doesn’t show any amino acid sequence identity with CXCR3 ligands, showed to activate this chemokine receptor. The 3D structure of CXCR3 is currently not known, but a general “two-step” model has been proposed to describe the chemokine receptor activation [45]. The first step is governed by binding of the ligand to the N-terminus and extracellular loops of the GPCR [46], subsequently, the N-terminus of the chemokine is able to interact with the transmembrane domains (TM), leading to activation of the receptor [45,46]. In contrast, it is likely that this two-step binding model cannot not be applied in the case of small molecule modulators, like PDT. Indeed, GPCRs possess multiple binding sites and different ligands can selectively stabilize different “active” conformations [47,48]. Small molecules, which don’t mimic the N-terminal regions of chemokines, rather point out other important interactions and generally bind to the TM part [49,50], as recently shown for example for a small molecule CXCR4 antagonist using X-ray crystallography [51]. In addition, Xanthou et al. (2003) did not validate the two-step model for CXCR3 interactions with its ligands, but instead support a multi-site model, in which multiple and distinct extracellular domains contribute to receptor activation [52]. Consequently, small molecule modulators are considered to allosterically modulate GPCR function from binding sites that are distinct or only partially overlapped with the chemokine interaction points [53,54,55,56]. Future studies on PDT structure−activity relationship will be necessary to reveal the modality of dipeptide to trigger CXCR3 and could be of both fundamental and practical relevance.

CXCR3 is known to play a key role in mediating leukocyte recruitment to mucosal tissues and inflammatory sites [57,58,59,60,61]. In particular, different studies demonstrated a key role for CXCR3 in recruitment, trafficking and function of Th1 CD4+ and effector CD8 T cells to infection sites and the establishment on Th1 amplification loop mediated by IFNγ and IFNγ-inducible CXCR3 ligands 61. Moreover, CXCR3 is known to play a role in the migration of T cells within peripheral tissue and lymphoid compartment, facilitating their interaction with antigen presenting cells and leading to the generation of effector, regulatory and memory T cells [61]. On the other side, it has been demonstrated that PDT is capable to enhance the proliferation of PBMCs and their production of crucial cytokines, as IFN-γ and IL-12 (20), to induce T cell migration (Figure 5E), drive T cell proliferation and differentiation towards a Th1 phenotype [16,19], and induce maturation of dendritic cells (DCs) [18]. In this regard, we can hypothesize that the dipeptide could attract T cells through the CXCR3 receptor, co-stimulate their proliferation, differentiation and activation, priming T cell responses with consequent therapeutic implications following local delivery. Moreover, like many chemokines, PDT might direct the migration of circulating leukocytes in the lymphoid tissues regulating and ensure continued recirculation and surveillance of lymphoid tissues. In particular, PDT administration in defined anatomically restricted locations (in vivo) might act as homing molecule in the control of T-cell trafficking over the course of immune response in mucosa-associated lymphoid tissues (MALTs). However, how PDT could be capable of coordinating T cell responses in the inflamed periphery through CXCR3 remains to be determined and future studies should be aimed at studying the role of this interaction in the control of T cell function.

Finally, since diverse studies suggested that CXCR3 activation could be beneficial in skin wound healing [62,63,64] and CXCR3 agonists possess antitumor activity [65,66,67], it might be interesting to evaluate the use of PDT in different pathological processes. Therefore, from a therapeutic point of view, our demonstration of a chemokine-like activity of PDT through CXCR3, will call for a future exploration of therapeutic potential of using this synthetic dipeptide in different diseases.

## 4. Materials and Methods

### 4.1. Pidotimod Preparation

PDT (purity > 99.6%) was a kind gift of Polichem SA (Lugano, CH). For all experiments the same PDT stock solution was used (10 mg/mL in ultrapure endotoxin-free water, stored in aliquots at −20 °C). Biological effects were evaluated using PDT at the final concentrations of 0.01, 0.05, 0.1, 0.5, 1, 5, 25, 50 and 100 μg/mL. The absence of endotoxin contamination (<0.25 unit/500 μg) in the PDT stock solution was assessed by Limulus amoebocyte assay (Associates of Cape Cod, East Falmouth, MA, USA) and all experiments were performed in endotoxin free-condition.

### 4.2. Cell Culture

The murine pre-B L1.2 cells were obtained from the American Type culture collection (Manassas, VA) and cultured in RPMI 1640 containing 10% fetal bovine serum (FBS, Lonza, Basel, CH), 2 mM L-glutamine, 1 mM sodium pyruvate and 50 μM 2-mercaptoethanol (complete medium).

### 4.3. Isolation of Human Primary Cells

Blood was collected from healthy donors who gave informed consent for this research according to the Declaration of Helsinki. PBMCs were isolated by using Ficoll-Paque (Sigma-Aldrich, St. Louis, MO, USA) density gradient centrifugation and then human primary monocytes were isolated from PBMCs by negative selection using the monocyte isolation Kit (Miltenyi Biotec, Bergish Gladbach, NRW, DA) according to the manufacturer’s protocol. Monocytes were cultured (2 × 10^6^/mL) in RPMI 1640 containing 10% fetal bovine serum (low endotoxin FBS, Lonza) and 2 mM L-glutamine (complete medium). In each experiment, cell purity was evaluated by flow cytometry and was always greater than 97%.

Human primary lymphocytes were isolated from PBMC by Percoll gradient sedimentation, as previously described [68]. T lymphocytes were isolated by negative selection (Miltenyi Biotec) according to manufacturer’s instructions. Purity of T lymphocyte preparation was evaluated by flow cytometry after staining with fluorochrome-conjugated anti-CD3 antibodies and was more than 95%. Isolated T lymphocytes were seeded (2 × 10^6^/mL) in 6 well plates and kept in culture for 6 days in complete medium supplemented with IL-2 (20 ng/mL) (R&D System, Minneapolis, MN, USA) to obtain activated T cells. After 3 days the medium was changed with complete medium containing the cytokine. After 6 days of culture, the cells were harvested, pooled together and counted for the assays.

Monocytes were pretreated for 2 h at 37 °C, when indicated, with PTx (500 ng/mL) or 30 min with LY294002 (25 μM) or wortmannin (100 nM) or Akt inhibitor VIII (1 μM) (Enzo Life Sciences, Farmingdale, NY, USA) or Tyrphostin AG490 (10 μM) (Vinci-Biochem, Firenze, IT) or PD98059 (10 μM) or staurosporine (1 nM) (Sigma-Aldrich) or AMG 487 (0.5 μM, a CXCR3 antagonist) (Tocris Bioscience, Bristol, GB) or with a control IgG antibody and a neutralizing mAb to CXCR1 (MAb330) or CXCR2 (MAb331) or CXCR3 (MAb160) or CXCR4 (MAb171) (50 μg/mL) (R&D System).

### 4.4. Chemotaxis Assays

Migration of monocytes, T lymphocytes and L1.2 cells was assessed using 5-μm pore-size Transwells (BD Biosciences, Franklin Lakes, NJ, USA). Chemotaxis assays were performed as previously described [27]. Briefly, cells, pre-treated or not with PTx or different inhibitors, were suspended at 2 × 10^6^/mL in adhesion buffer. Cell suspension (100 μL) was added to the top well and adhesion buffer (600 μL) containing Pidotimod (0.01, 0.05, 0.1, 0.5, 1, 5, 25 and 50 μg/mL) to the bottom well. LPC (L-α-lysophosphatidylcholine, palmitoyl C16:0, Sigma-Aldrich) (10 µM) and fMLP (10 nM) were also used as reference chemoattractants to evaluate the selective and not toxic effect of PTx; CXCL8, CXCL11 and CXCL12 (R&D System) (10 nM) were used as chemokine ligands to evaluate the specificity of antibodies. Chemotaxis was performed for 90 min at 37 °C and then filters were removed. After fixation with 1.5% paraformaldehyde, migrated cells were counted in 5 high-power fields by light microscopy at a 10× magnification. Results are expressed as the fold increase compared with control.

### 4.5. Static Adhesion Assay

Adhesion assays were performed as previously described [27]. Monocytes, pre-treated or not with PTx, were suspended at 5 × 10^6^/mL in adhesion buffer (PBS, 1 mM CaCl_2_, 1 mM MgCl_2_, 10% FCS, pH 7.2) and 20 μL of cell suspension was added to 18-well glass slides (Thermo Fisher Scientific, Waltham, MA, USA) coated overnight at 4 °C with ICAM-1 and V-CAM (1 μg/mL, R&D Systems). Cells were then stimulated for 2 min at 37 °C with 5 μL of PDT at different concentrations (1, 5, 10, 50, 100 μg/mL). PMA (phorbol 12-myristate 13-acetate) stimulation (100 ng/mL) at 37 °C for 10 min was also evaluated to exclude a toxic effect of PTx. After washing, adherent cells were fixed in 1.5% glutaraldehyde. Computer-assisted enumeration of cells in 4 high-power fields by light microscopy was performed. Results are expressed as the -fold increase compared with control.

### 4.6. Western Blot Analysis

Monocytes (10 × 10^6^/mL) were stimulated with fMLP (10 nM) and PDT at different concentrations 1, 5, 25, 50, 100 μg/mL and then lysed in 200 μL of buffer (pH 7.2) containing 20 mM MOPS (pH 7.0), 2 mM EGTA, 5 mM EDTA, 30 mM sodium fluoride, 60 mM glycerophosphate (pH 7.2), 20 mM sodium pyrophosphate, 1 mM sodium orthovanadate, 1 mM Dithiothreitol (DTT), 1% Triton X-100 and a mixture of protease inhibitors (Complete Mini Roche, Hoffmann-La Roche, Basel, CH). The total concentration of proteins was detected by QuantiProTM BCA Assay (Sigma-Aldrich). Equal amounts of total protein were resolved on a 12% SDS-polyacrylamide gel and then electroblotted using PVDF (polyvinylidene difluoride) membrane. The blots were incubated overnight at 4 °C with 1) mouse monoclonal p-Tyr antibody (PY99) (Santa Cruz Biotechnology, Dallas, TX, USA), 2) rabbit polyclonal phospho-Akt (Ser473) antibody (Santa Cruz Biotecnology), 3) mouse monoclonal Akt antibody (Cell Signaling Technology, Danvers, MA, USA), 4) rabbit polyclonal antibody to ERK1/2 (Santa Cruz Biotechnology), 5) mouse monoclonal GADPH antibody (Santa Cruz Biotechnology). Antigen-antibody complexes were revealed by incubating the membranes at room temperature for 1h with peroxidase-conjugated anti-mouse or anti-rabbit antibodies (Thermo Fisher Scientific) and using the ECL (Enhanced Chemiluminescence) System (Santa Cruz Biotechnology). The images were captured by ChemiDoc-It Imaging System and the integrated optical density (IOD) was determined using the Gel-Pro Analyzer 6.0 software (Houston, TX, USA.

### 4.7. siRNA Technique

Monocytes were nucleoporated (program Y-001) with specific siRNAs for CXCR3 receptor (Origene, Rockville, MD, USA) using the Amaxa Nucleofector System (Amaxa Biosystems, Cologne, NW, DE). In particular, siRNAs (100 nM) were added to 3 × 10^6^ cells resuspended in 100 mL of nucleofection buffer. Fluorescein-labeled irrelevant (scr) siRNAs (Invitrogen, Carisbad, CA, USA) were used as negative control and to assess the efficiency of siRNA nucleoporation by flow cytometry. The efficacy of CXCR3 siRNA was evaluated by real-time PCR analysis.

### 4.8. Real-Time PCR for Gene Expression Analysis

Total RNA was isolated from monocytes (1 × 10^6^ cells) using RNeasy Plus Mini Kit (Qiagen, Valencia, CA, USA). Following retrotranscription, 50 ng of cDNA mixed with sterile water and SYBR Green qPCR Master Mix (Promega, Madison, WI, USA) were amplified using the PrimeTime qPCR primer Assay for CXCR3 and the following PCR primers (0.2 μM each): human β actin, 5′- GGCACCCAGCACAATGAAG -3′ (forward), and 5′- GCTGATCCACATCTGCTGG -3′ (reverse) (Integrated DNA technologies, Coralville, IA, USA). Quantification of CXCR3 cDNA was normalized in each reaction according to the internal β-actin control. Results are expressed as percentage of control.

### 4.9. Cell Viability Assay

Cell viability was evaluated according to the manufacturer’s instructions by CellTiter-Glo Luminescent Cell Viability Assay (Promega), which is based on the quantification of ATP. The same number of treated (PDT 5, 25, 100 μg/mL) and not treated cells for 120 min were analyzed.

### 4.10. Flow Cytometry Analysis

Cell integrity was analyzed by flow cytometry. In brief, treated (PDT 5, 25, 100 μg/mL) or not treated cells with PDT were harvested, washed with phosphate-buffered saline (PBS) and pelleted by centrifugation. Then, the cells were stained for nuclear DNA content with 50 μg/mL propidium iodide (PI) (Sigma-Aldrich). Determination of PI-stained cells was performed in FL-2A channel on FACSCalibur flow cytometer and data were acquired and analyzed by using CellQuest Software (BD Bioscience).

### 4.11. Transfections

Murine pre-B cells L1.2 were cultured in RPMI 1640 containing 1 mM L-glutamine, 1 mM sodium pyruvate, 10% FBS and 50 μM 2-mercaptoethanol. Transfections of L1.2 cells were performed using Amaxa nucleofector (Lonza) (program U-015), according to the manufacturer’s instructions. Cells were plated at 1 × 10^6^/mL and, after 24 h, 4 × 10^6^ cells were nucleofected with 3 mg endotoxin-free plasmids pTarget or pTarget-CXCR3A. The vector pTarget was from Promega and was used as negative control. In pTarget-CXCR3A construct (a kind gift from Alan Wells, Department of Pathology, University of Pittsburgh, Pittsburgh, Pennsylvania, USA) CXCR3A (NM_001504.1) was cloned into the EcoRI and SalI sites of pTarget. After 4 h from transfection, in order to enhance CXCR3A cell surface expression, transient transfectants were incubated overnight at 37 °C in medium supplemented with 10 mM sodium butyrate (Sigma-Aldrich). The transfection efficiency was evaluated as GFP expression by flow cytometry using control vector pmaxGFP included in Amaxa nucleofector kit. Cell surface expression of CXCR3A was evaluated by flow cytometry using an anti-human CXCR3 PE-conjugated mAb (Thermo Fisher Scientific).

### 4.12. Statistical Analysis

Data obtained from multiple independent experiments are expressed as the means ± the standard deviations (SD). The data were analyzed for statistical significance using a paired 2-tail Student *t* test or one-way ANOVA. Bonferroni’s post-test was used to compare data. Differences were considered significant at *p* < 0.05. Statistical tests were performed using Prism 5 software (GraphPad Software, La Jolla, CA, USA).

## Supplementary Materials

Supplementary materials can be found at https://www.mdpi.com/1422-0067/20/21/5287/s1.
