# Supplementary material for: The Synthetic Dipeptide Pidotimod Shows a Chemokine-Like Activity through CXC Chemokine Receptor 3 (CXCR3)
Source: Int J Mol Sci. 2019 Oct 24;20(21):5287. doi: 10.3390/ijms20215287 (PMC6862300; doi:10.3390/ijms20215287)
Supplement: Supplementary file 1 [file ijms-20-05287-s001.pdf]

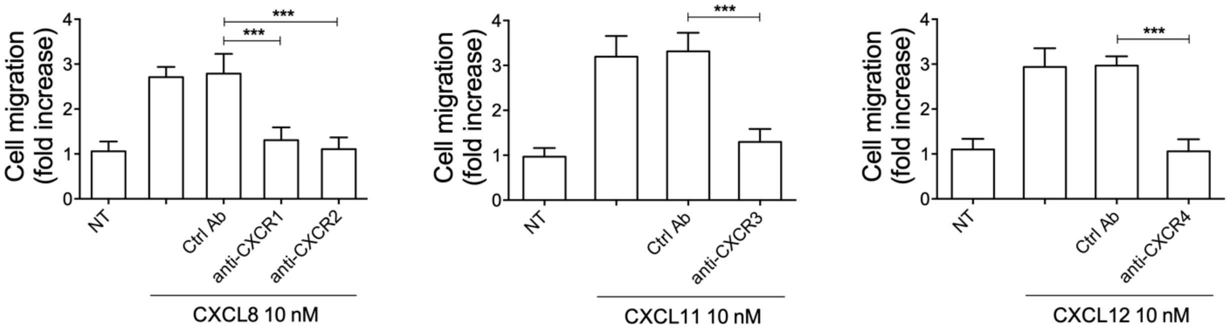

Figure S1. Specificity of CXCR1, CXCR2, CXCR3 and CXCR4 neutralizing antibodies. Transwell migration assay of monocytes in response to the indicated treatments. Monocytes pretreated for 1 h at 37 °C with 50 μg/mL of Ctrl mAb or mAb to anti-CXCR1 or anti-CXCR2 or anti-CXCR3 or anti-CXCR4 were stimulated for 90 min at 37 °C with PBS (NT) or CXCL8 or CXCL11 or CXCL12 (10 nM). Bars represent the mean ± SD of two independent experiments performed in triplicate. Statistical analysis was performed by one-way ANOVA and the Bonferroni's post-test was used to compare data. *** $p < 0.001$. NT = not treated.
